# Triglyceride-glucose index predicts all-cause mortality, but not cardiovascular mortality, in rural Northeast Chinese patients with metabolic syndrome: a community-based retrospective cohort study

**DOI:** 10.1186/s12986-024-00804-0

**Published:** 2024-05-21

**Authors:** Shasha Yu, Qiyu Li, Hongmei Yang, Xiaofan Guo, GuangXiao Li, Yingxian Sun

**Affiliations:** 1 Department of Cardiology, First Hospital of China Medical University, 155 Nanjing North Street, Heping District, Shenyang, 110001 China; 2 Department of Clinical Epidemiology, Institute of Cardiovascular Diseases, First Hospital of China Medical University, Shenyang, 110001 China

**Keywords:** Metabolic syndrome, Triglyceride-glucose index, Cardiovascular mortality, All-cause mortality, Rural China

## Abstract

**Background:**

Metabolic syndrome (MetS) includes a group of metabolic irregularities, including insulin resistance (IR), atherogenic dyslipidemia, central obesity, and hypertension. Consistent evidence supports IR and ongoing low-grade inflammation as the main contributors to MetS pathogenesis. However, the association between the triglyceride-glucose (TyG) index and mortality in people with MetS remains uncertain. The objective of this study was to examine the correlation between the baseline TyG index and all-cause and cardiovascular (CV) mortality in rural Northeast Chinese individuals with MetS.

**Methods:**

For the Northeast China Rural Cardiovascular Health Study, 3918 participants (mean age, 55 ± 10; 62.4% women) with MetS at baseline were enrolled in 2012–2013 and followed up from 2015 to 2017. The TyG index was calculated using the equation TyG index = ln [fasting TG (mg/dL) × fasting glucose (mg/dL)/2] and subdivided into tertiles [Q1(< 8.92); Q2 (8.92–9.36); Q3 (≥ 9.36)]. Multivariate Cox proportional hazards models were developed to examine the correlations between mortality and the baseline TyG index.

**Results:**

During a median of 4.66 years of follow-up, 196 (5.0%) all-cause deaths and 108 (2.8%) CV disease-related deaths occurred. The incidence of all-cause mortality was significantly different among TyG index tertiles of the overall population (*P* = 0.045). Kaplan–Meier analysis demonstrated a significantly increased risk of all-cause mortality in rural Chinese patients with a higher TyG index (log-rank *P* < 0.05). After adjusting for possible confounders, Cox proportional hazard analysis revealed that the TyG index could effectively predict all-cause mortality (HR for the third vs. first tertile of TyG was 1.441 [95% confidence interval, 1.009–2.059]), but not CV mortality, in rural Chinese patients with MetS.

**Conclusions:**

The TyG index is an effective predictor of all-cause mortality in rural Chinese patients with MetS. This indicates that the TyG index may be useful for identifying rural Chinese individuals with MetS at a high risk of death.

## Introduction

Metabolic syndrome (MetS) includes a cluster of conditions associated with metabolic dysregulation, such as insulin resistance (IR), atherogenic dyslipidemia, central obesity, and hypertension. Cumulative evidence has confirmed that IR and persistent low-grade inflammation are the primary pathogenic factors of MetS [1]. SPECT-China, a population-based cross-sectional survey of Chinese individuals ≥ 18 years of age, reveals a 22.0% age-standardized prevalence of MetS in East China [2]. Similarly, our previous study found that MetS has gradually become more prevalent among participants from rural China (39.0%) [3]. If left untreated, MetS considerably increases morbidity and mortality [1]. It is thus crucial to investigate an effective predictor of mortality in patients with MetS to reduce the substantial disease burden.

Recently, the triglyceride-glucose (TyG) index has garnered increasing global attention, as it is associated with all-cause and cardiovascular (CV) mortality in the general population [4]. Similarly, Liao et al. observed a strong association between the TyG index and an increase in all-cause mortality in critically ill patients [5]. A U-shaped association was further observed between the TyG index and all-cause mortality in American patients with cardiovascular diseases (CVDs) and diabetes or prediabetes [6]. Multiple studies have also comprehensively investigated the predictive value of the TyG index as a biomarker for IR [7]. As the underlying mechanism of MetS is IR, it can also be diagnosed by measuring the TyG index [8]. However, whether the TyG index can predict mortality among patients with MetS remains unknown.

As previously mentioned, economic development has led to increasing trends in metabolic disorders in rural Chinese areas. The TyG index is affordable and readily available, making it a suitable and widely used index to predict mortality among patients with MetS in rural areas. The aim of this study was to evaluate the impact of the baseline TyG index on all-cause and CV mortality in participants with MetS from rural areas of China.

## Methods

### Study population

**Fig. 1:**
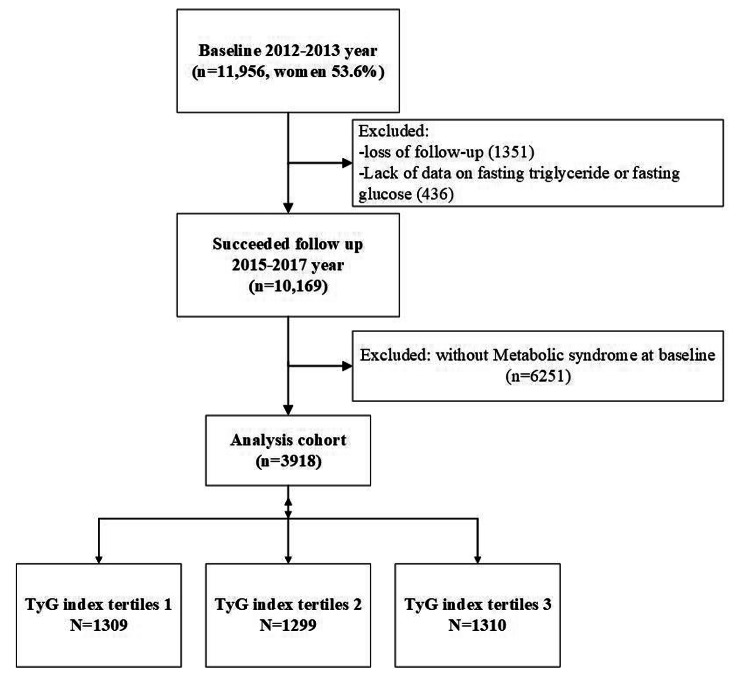
Process for the inclusion of trial patients. TyG index, triglyceride-glucose index

A community-based retrospective cohort study was carried out in rural Northeast China, with the study details previously described [3]. This research was granted approval by China Medical University’s Ethics Committee (Shenyang, China AF-SDP-07-1, 0–01). Baseline information was acquired from the 2012–2013 survey, and 11,956 individuals were enrolled. The study cohort was subjected to follow-up from 2015 to 2017, with a median duration of 4.66 years. The comprehensive procedure for participant inclusion is presented in Fig. 1, resulting in data analysis for 3918 participants.

### Study variables

The participants’ heights and weights were recorded while wearing casual clothing and not wearing shoes. At the umbilicus, the waist circumference was measured using non-elastic tape. The participants’ blood pressure was measured three times using an electronic, standardized, automated manometer (HEM-907; Omron, Tokyo, Japan) after at least 5 min of sitting still. Participants’ blood was extracted after a fasting period of at least 12 h, and enzymatic methods were used to identify the fasting plasma glucose (FPG), low-density lipoprotein cholesterol (LDL-C), and other biochemical indicators that are frequently evaluated.

**Fig. 2:**
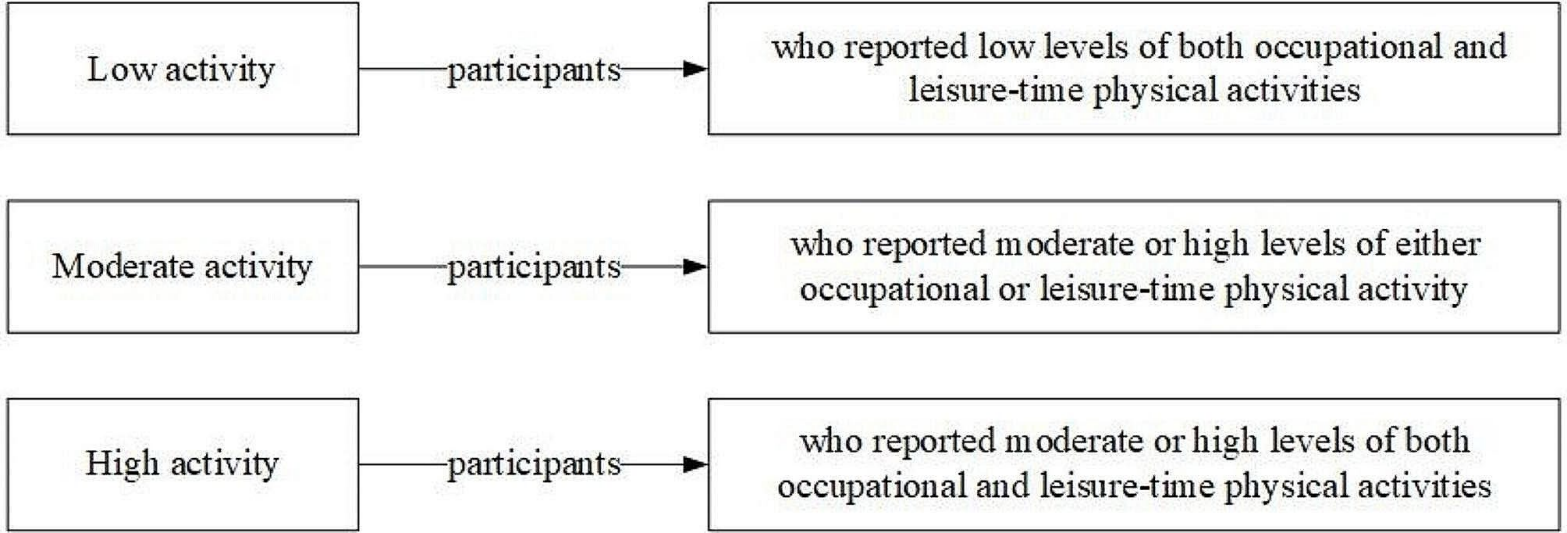
Categories of physical activity

At baseline, a standardized questionnaire was used in an interview to obtain information on medical histories, lifestyle factors, and demographic features. Additionally, current alcohol and tobacco use was defined. The total duration of sleeping (in hours) across the span of 24 h was utilized for calculating the duration of sleep. An individual’s educational situation was ascertained according to the completion of elementary, middle, or high school. The family’s annual income was categorized as follows: ≤5000 CNY (788 dollars), 5000–20,000 CNY (788–3152 dollars), or > 20,000 CNY (3152 dollars). The physical activity of the participants (Fig. 2), encompassing both work-related and recreational pursuits, was evaluated and divided into three categories [3].

### Definition

**Fig. 3:**
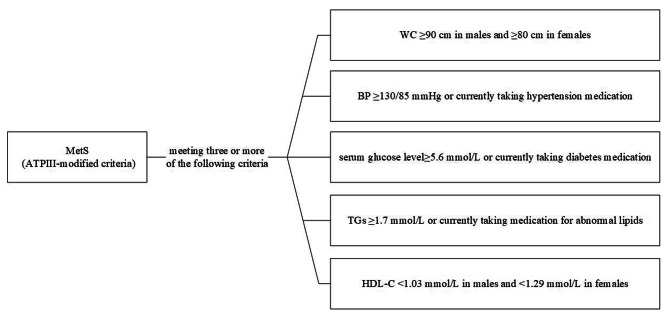
Definition of metabolic syndrome and metabolic disorders

The TyG index was calculated using the following equation: ln [fasting triglycerides (mg/dL) × fasting glucose (mg/dL)/2] [9]. Body mass index (BMI) was determine using the following equation: BMI = $\frac{weight \left(kg\right)}{{\left(height\right)}^{2} \left(m\right)}$. Figure 3 provides the definition of the ATPIII-modified criteria [10].

### Statistical analyses

The individuals were divided into three groups based on their TyG index levels, with each group representing one-third of the total participants, as follows: tertile 1 (*n* = 1309, TyG index < 8.92), tertile 2 (*n* = 1299, 8.92 ≤ TyG index < 8.92–9.36), and tertile 3 (*n* = 1310, TyG index ≥ 9.36), with the depicted characteristics. Categorical variables have been quantified using numerical values (n) and percentages (%) and were evaluated using the chi-squared test. Continuous variables with a normal distribution are expressed as the mean ± standard deviation; non-normal distributions are represented as the median (interquartile range). Data were analyzed using one-way analysis of variance for a normal distribution and the Kruskal–Wallis test for a non-normal distribution. We investigated the precise correlation between the TyG index and mortality from all causes and CVDs in people with MetS using multivariate Cox proportional hazards models. Three sets of models were constructed in this study. Model 1 included only the TyG index, whereas Model 2 incorporated demographic attributes such as age, sex, and race. Model 3 further adjusted for education, current smoking and drinking habits, annual income, sleep duration, physical activity, and cardiovascular history. A stratified analysis was conducted to examine the impact of putative effect modifiers, including age, sex, BMI, and current smoking and drinking status, on relevant variables. The statistical analyses were conducted using R software (version 4.2.1; R Foundation for Statistical Computing, Vienna, Austria), and statistical significance was defined as *P* < 0.05.

## Results

### Population characteristics stratified using the TyG index

As presented in Table 1, baseline participant characteristics were stratified using TyG index tertiles (Q) as follows: Q1, < 8.92; Q2, 8.92–9.36, and Q3 ≥ 9.36. The mean TyG index levels in these tertiles were 8.54 ± 0.29, 9.13 ± 0.122, and 9.87 ± 0.48, respectively. The participants with higher TyG index values were generally male, older, and current smokers and drinkers, had a longer sleep duration, and exhibited a higher prevalence of CV comorbidities. Additionally, they experienced a higher incidence of all-cause mortality than those with lower TyG index values (4.1% vs. 4.7% vs. 6.2%, *P* = 0.045). Moreover, the higher TyG index group exhibited higher levels of HbA1C, LDL-C, TC, TG, uric acid, FPG, AST, and ALT, along with lower eGFR and HDL-C levels (Table 2).

**Table 1:** Baseline characteristics of study participants by Triglyceride glucose Index (TyG) index tertile

Variables	Tertiles of TyG index				
	overall	Q 1(< 8.92)	Q 2 (8.92–9.36)	Q 3(≥ 9.36)	*P* value
**N**	3918	1309	1299	1310	
**Gender (female)**		935(71.4)	779(60.0)	730(55.7)	< 0.001
**Age (years)**	55.44 ± 10.18	54.77 ± 10.44	56.02 ± 10.26	55.53 ± 9.80	< 0.001
**Current smoking (yes)**	1170(29.9)	299(22.8)	425(32.7)	446(34.0)	< 0.001
**Current drinking (No)**	706(18.0)	170(13.0)	233(17.9)	303(23.1)	< 0.001
**Ethnicity** ^**a**^ **(Han)**	3705(94.6)	1235(94.3)	1243(95.7)	1227(93.7)	0.068
**Education status**					0.126
Primary school or below	2174(55.5)	692(52.9)	752(57.9)	730(55.7)	
Middle school	1366(34.9)	480(36.7)	434(33.4)	452(34.5)	
High school or above	378(9.6)	137(10.5)	113(8.7)	128(9.8)	
**Annual income (CNY/year)**					0.708
≤ 5000	481(12.3)	154(11.8)	154(11.9)	173(13.2)	
5000–20,000	2134(54.5)	709(54.2)	710(54.7)	715(54.6)	
> 20,000	1301(33.2)	445(34.0)	435(33.5)	421(32.2)	
**Sleep duration (h/d)**					0.017
≤7	1968(50.3)	657(50.2)	676(52.2)	635(48.5)	
7–8	1060(27.1)	373(28.5)	349(26.9)	338(25.8)	
8–9	558(14.3)	189(14.4)	163(12.6)	206(15.7)	
>9	326(8.3)	89(6.8)	108(8.3)	129(9.9)	
**Physical activity**					0.276
Low	1680(43.2)	530(40.8)	574(44.4)	576(44.4)	
Moderate	712(18.3)	244(18.8)	228(17.6)	240(18.5)	
High	1494(38.4)	524(40.4)	490(37.9)	480(37.0)	
**Pulse (times/min)**	81 ± 14	79 ± 13	80 ± 14	83 ± 14	< 0.001
**BMI (kg/m**^**2**^)	26.89 ± 3.39	26.84 ± 3.39	26.65 ± 3.36	27.19 ± 3.41	< 0.001
**WC (cm)**	88.54 ± 8.67	88.38 ± 8.39	87.71 ± 9.17	89.52 ± 8.35	< 0.001
**Height (m)**	159.95 ± 8.36	159.28 ± 7.94	160.09 ± 8.30	160.48 ± 8.78	0.001
**SBP (mmHg)**	151.10 ± 22.71	150.37 ± 22.27	150.06 ± 22.86	152.87 ± 22.92	0.003
**DBP (mmHg)**	86.26 ± 11.53	85.52 ± 11.21	85.99 ± 11.38	87.25 ± 11.93	< 0.001
**Cardiovascular Comorbidities (Yes)**	448(11.4)	138(10.5)	129(9.9)	181(13.8)	0.004
**Out comes**					
All-cause mortality, n (%)	196(5.0)	54(4.1)	61(4.7)	81(6.2)	0.045
Cardiovascular mortality, n (%)	108(2.8)	29(2.2)	37(2.8)	42(3.2)	0.293

^a^ others including some ethnic minorities in China, such as Mongol and Manchu. Abbreviations: CNY China Yuan (1CNY = 0.161 USD), BMI body mass index, WC waist circumference, SBP Systolic Blood pressure, DBP diastolic blood pressure, FPG fasting plasma glucose, HDL-C high-density lipoprotein, LDL-C low-density lipoprotein cholesterol

**Table 2:** Baseline levels of laboratory characteristics according to the Triglyceride glucose Index (TyG) index quartiles

Variables	Tertiles of TyG index				
	overall	Q 1(< 8.92)	Q 2 (8.92–9.36)	Q 3(≥ 9.36)	*P* value
HbA1C(%)	5.67 ± 1.16	5.39 ± 0.69	5.49 ± 0.82	6.12 ± 1.56	< 0.001
eGFR(ml/min/1.73m^2^)	90.23 ± 16.02	91.57 ± 15.37	89.41 ± 16.54	89.70 ± 16.07	0.001
LDL-C(mmol/L)	3.18 ± 0.31	2.98 ± 0.76	3.27 ± 0.88	3.29 ± 0.99	< 0.001
HDL-C (mmol/L)	1.25 ± 0.31	1.31 ± 0.34	1.24 ± 0.29	1.19 ± 0.28	< 0.001
TC (mmol/L)	5.49 ± 1.18	5.06 ± 0.99	5.51 ± 1.06	5.92 ± 1.30	< 0.001
TG(mmol/L)	2.33 ± 1.92	1.15 ± 0.33	1.98 ± 0.36	3.85 ± 2.63	< 0.001
Uric acid(umol/L)	306.22 ± 87.99	282.37 ± 77.61	310.59 ± 87.49	324.71 ± 93.14	< 0.001
FPG (mmol/L)	6.50 ± 2.07	5.92 ± 0.88	6.04 ± 1.14	7.54 ± 3.03	< 0.001
BUN(mmol/L)	5.55 ± 2.48	5.64 ± 3.06	5.53 ± 2.08	5.47 ± 2.18	0.222
AST (IU/L)	22.46 ± 11.24	21.33 ± 9.96	22.38 ± 9.59	23.68 ± 13.60	< 0.001
ALT(IU/L)	25.12 ± 17.51	21.85 ± 14.98	25.16 ± 16.36	28.36 ± 20.17	< 0.001

Date are presented as mean (SD) or n (%);

### Associations between the TyG index and all‑cause and CV mortality

Cox proportional hazard analysis revealed a significant association between the TyG index and all-cause mortality, but not CV mortality, in both crude and adjusted models [crude: all-cause mortality HR, 1.303 (95% CI, 1.061–1.600), *P* = 0.012; CV mortality HR, 1.224 (95% CI, 0.923–1.623), *P* = 0.160]; Model 2: all-cause mortality HR, 1.330 (95% CI, 1.075–1.646), *P* = 0.009; CV mortality HR, 1.249 (95% CI, 0.931–1.674), *P* = 0.138; Model 3: all-cause mortality HR, 1.288 (1.033–1.605), *P* = 0.025; CV mortality HR, 1.194 (95% CI, 0.879–1.622), *P* = 0.257]. In both the crude and adjusted models, upward trends were observed between the TyG index and all-cause mortality (Table 3, both *P* < 0.05). The participants in the Q3 TyG index tertile had a significantly higher incidence of all-cause mortality than those in the Q1 and Q2 tertiles [HR, 1.441 (95% CI, 1.009–2.059), *P* = 0.016].

**Table 3:** HRs (95% CIs) for mortality according to the triglyceride glucose index (TyG) index tertiles

	Tertiles of TyG index			
	Q 1(< 8.92)	Q 2 (8.92–9.36)	Q 3(≥ 9.36)	*P* trend
**All-cause mortality**				
**Number of deaths**	54	61	81	
Model 1				
HR(95%CI) *P*-value	1	1.147(0.795,1.654) 0.464	**1.496(1.060,2.111)** **0.022**	**0.020**
Model 2				
HR(95%CI) *P*-value	1	1.036(0.718,1.495) 0.851	**1.498(1.061,2.115)** **0.021**	**0.018**
Model 3				
HR(95%CI) *P*-value	1	1.100(0.758,1.599) 0.615	**1.441(1.009,2.059)** **0.045**	**0.016**
**CVD mortality**				
**Number of deaths**	29	37	42	
Model 1				
HR(95%CI) *P*-value	1	1.296(0.797,2.106) 0.297	1.446(0.901,2.321) 0.127	0.129
Model 2				
HR(95%CI) *P*-value	1	1.148(0.706,1.868) 0.578	1.455(0.905,2.337) 0.121	0.116
Model 3				
HR(95%CI) *P*-value	1	1.277(0.772,2.113) 0.340	1.403(0.851,2.313) 0.184	0.108

Model 1: Non-adjusted

Model 2: Adjusted for age, race and gender

Model 3: Adjusted for age, gender, race, BMI, SBP, DBP, current smoking and drinking, education, annual income, sleep duration, physical activity, cardiovascular history

HR: Hazard ratio; CI: Confidence interval

### Subgroup analysis of the association between the TyG index and all‑cause and CV mortality

**Fig. 4:**
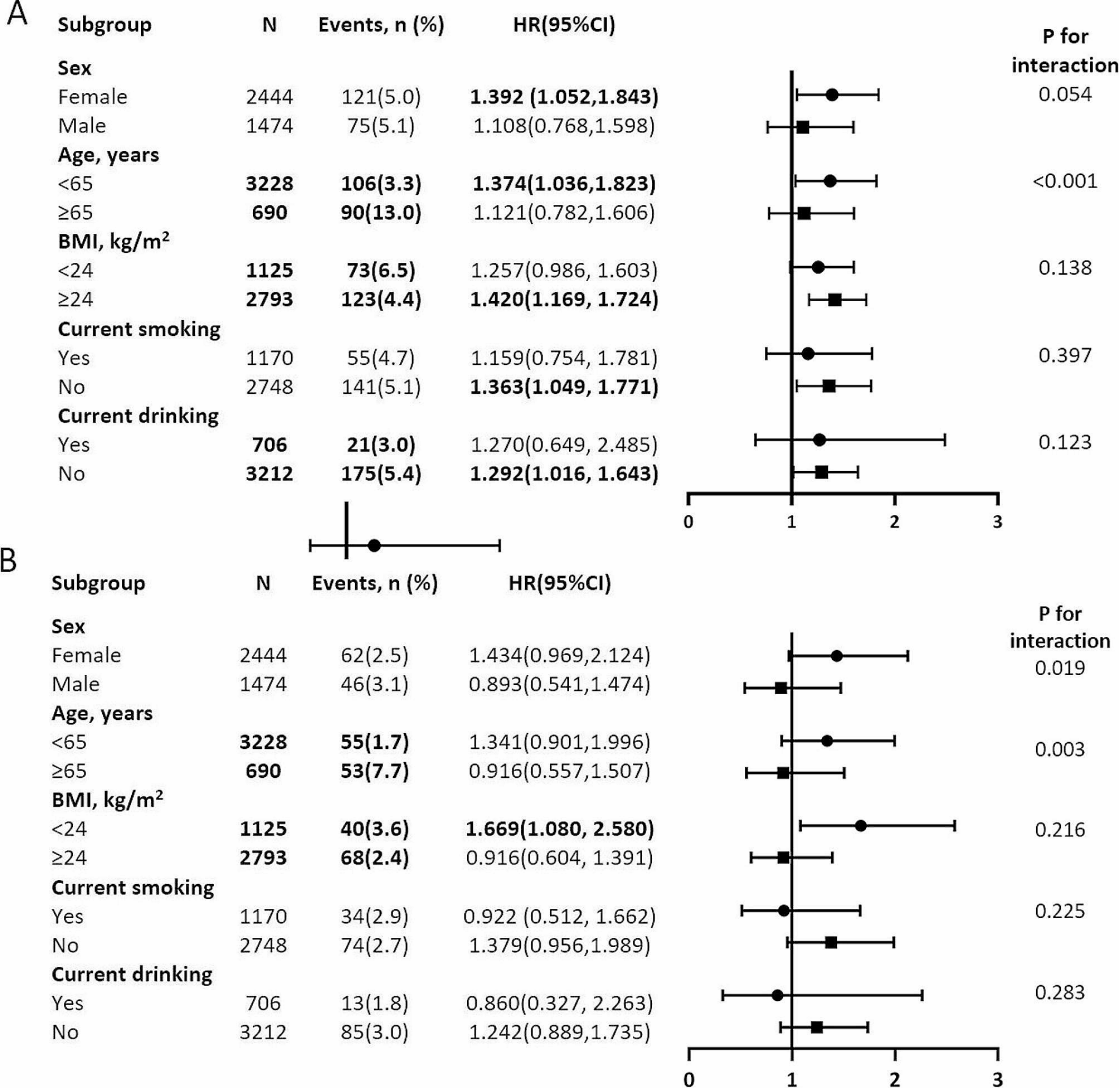
Subgroup analysis of the association between Triglyceride glucose Index (TyG) index and all-cause mortality (**A**), cardiovascular disease (CV) mortality (**B**) among rural Chinese with Metabolic syndrome (MetS). Each subgroup analysis is adjusted if not stratified for age, gender, race, BMI, SBP, DBP, current smoking and drinking, education, annual income, sleep duration, physical activity, cardiovascular history

Stratification was performed based on age, sex, BMI, and current smoking and drinking status to evaluate the impact of the TyG index on the primary endpoints (Fig. 4). Except for the age subgroup (age subgroup: all-cause mortality, P for interaction < 0.001), no significant interactions were observed in most subgroups. The TyG index was associated with all-cause mortality in patients < 65 years of age [all-cause mortality: HR, 1.374 (95% CI, 1.036–1.823)]; however, this was not the case for participants ≥ 65 years of age [all-cause mortality: HR, 1.121 (95%, CI 0.782–1.606)].

## Discussion

In this study, a substantial relationship between the TyG index and all-cause mortality, but not CV mortality, was found in a rural Chinese population with MetS. Moreover, a significant interaction effect was identified between age and the TyG index, suggesting that the correlation between TyG levels and mortality was particularly pronounced among patients who were younger in age. Recently, the incidence of MetS has considerably increased in rural Chinese areas. A meta-analysis indicated a 24.5% prevalence of MetS among individuals 15 years and older in mainland China, with 19.2% residing in rural areas [11]. Our previous study held in rural Northeast China revealed a cumulative incidence of newly diagnosed MetS at 24.0% [12]. Given that metabolic disorders often increase insidiously, all-cause and CV mortalities increase at the time of detection in this population [13]. Hence, proactive strategies and tasks should be undertaken to prevent risk factors such as hypertension, hyperlipidemia, elevated blood glucose, and obesity, along with effective measures to predict mortality among patients with MetS. IR is a crucial pathological factor in MetS, contributing to CVDs and poor clinical outcomes in various ways via mechanisms such as endothelial dysfunction, low-level inflammation, and disruptions in systemic glucose–lipid metabolism [14]. Although various indicators are used to measure IR, including the hyperinsulinemic euglycemic (HIEG) clamp test, homeostatic model assessment for insulin resistance (HOMA-IR), and quantitative insulin sensitivity check index, most of these indicators are time-consuming and expensive to determine, limiting their use in rural areas. Consequently, the TyG index has gradually gained attention in recent years owing to its cost-effectiveness compared to other parameters. In comparison to the gold standard (HIEG clamp test), Won et al. demonstrated that the TyG index has high sensitivity (96.5%) and specificity (85.0%) for IR detection [15]. Furthermore, when compared to the HOMA-IR, the TyG index was confirmed to perform better in assessing IR [16]. The precise mechanism explaining the connection between the TyG index and mortality is still not understood. Owing to the close relationship between the TyG index and IR, possible explanations could be related to IR-induced pathological processes. An imbalance in glucose metabolism may also be induced by IR, contributing to hyperglycemia, which, consequently, triggers inflammation and oxidative stress. Sasaki et al. found that oxidative stress and inflammation are effective predictors of mortality in patients undergoing hemodialysis [17]. Additionally, IR plays an important role in hyperlipidemia, which is a leading cause of CVDs, resulting in high mortality [18].

A correlation between the TyG index and increased all-cause mortality among rural Chinese individuals with MetS was confirmed for the first time in our study. The TyG index exhibits a notable correlation with an elevated susceptibility to metabolic diseases, such as hypertension [19, 20], diabetes [21], hyperuricemia [22], and MetS. Additionally, it is significantly associated with an increasing risk of various CVDs, including arterial stiffness, stroke, carotid atherosclerosis, myocardial infarction coronary artery disease, and peripheral artery disease [23–26]. Despite its effectiveness in predicting CVDs, the association between TyG and mortality remains inconsistent. A recent systematic review and meta-analysis, composed of 12 cohort studies with 6,354,990 participants, found no correlation between the TyG index and mortality in the overall population, including both cardiovascular and all-cause mortality [27]. Moreover, the correlation between TyG and mortality lost significance after adjusting for age, BMI, blood pressure, and smoking status in one study, whereas another study claimed a positive correlation [28, 29]. In contrast to the uncertain relationship between TyG and mortality in the general population, studies focusing on specific diseases provide a consistent conclusion. Shen et al. found that the TyG index is a predictive measure for the probability of death from any cause in older adults with acute coronary syndrome who also have diabetes [30]. Similar to that in older and middle-aged patients with type 2 diabetes, the TyG index may predict the likelihood of CVD-related mortality or death from any cause [31]. In participants with CVDs, the TyG index exhibited a U-shaped association with the risk of all-cause mortality [32]. Zhou et al. discovered a strong correlation between the TyG index and the mortality risk in individuals with chronic heart failure [33]. One reason for the discrepancy in the association between TyG and mortality could be the insufficient follow-up time. Furthermore, baseline accompanying diseases may influence the relationship between TyG and mortality.

One interesting finding in the present study was that the TyG index, a well-known parameter representing IR, was not significantly associated with cardiovascular mortality among rural Chinese individuals with MetS. The possible reasons for this contradiction might be as follows. First, the TyG index was first implemented in 2008 with the rationale that IR is frequently the reason for elevated TG and glucose levels in healthy individuals [9]. Not all studies found significant associations between TyG and CVD, especially among those accompanied by chronic diseases, such as diabetes and dyslipidemia [34, 35]. Moreover, the effect of triglycerides or glucose on cardiovascular events could be eliminated by antihyperlipidemic treatment and hypoglycemic drugs with CVDs [32]. The application of the TyG index for cardiovascular mortality can be also affected by hyperlipidemia and diabetes [9]. To justify the value of the TyG index as a biomarker, hypertriglyceridemia and glucose metabolic disorder should be well controlled. However, in the present study, we intended to estimate this relationship in Chinese patients with MetS, as our previous data indicated that high TG levels (32.1%) and increased fasting glucose (47.1%) was prevalent in rural Northeast Chinese individuals [12]. However, despite its high prevalence, its treatment and control rates are obviously low. Owing to the combined effect of these factors, it was not possible to investigate causation when applying the TyG index to these patients. Second, cardiovascular mortality was age-specific. Over 50% of the cardiovascular deaths among CVD patients comprised older individuals over 75 years of age [6]. Zhao et al. revealed a strong correlation between the TyG index and all-cause/CVD mortality in Americans with diabetes older than 65 years [31]. However, in our study, 82.7% of the participants were younger than 65 years, which might partially explain why the TyG index was not significantly correlated with cardiovascular mortality. Third, this also might be related to the relatively lower value of TyG index in present study. Zhou et al. found that only with a TyG index more than 9.52 will the risk for cardiovascular mortality increase [36]. However, in our study, the third tiles cut-off value of the TyG index was 9.36, which was relatively lower than those of previous studies [6, 37]. Fourth, the TyG index is susceptible to many confounding factors, including the existence of different ailments, the concomitant nutritional status, and altered blood lipid profiles. In our study, the coexisting MetS might have affected the relationship between the TyG index and cardiovascular mortality. Finally, heterogeneity in the study population and the insufficient follow-up time might have affected the relationship between the TyG index and cardiovascular mortality. Overall, more prospective studies are needed to verify the relationship between the TyG index and mortality.

Regarding participants with MetS at baseline, our study suggested that the TyG index can be a useful predictor of all-cause mortality. In rural areas, measuring triglyceride and glucose levels is affordable and easily accessible, with no apparent increase in participant and overall healthcare expenses with the TyG index determination. Previous studies support the TyG index as a reliable indicator for measuring IR and predicting mortality in populations with MetS. Furthermore, this approach may be practical and pragmatic for the large-scale screening of metabolic disorders, particularly in developing countries [7]. The merits of our investigation encompassed a substantial sample size and a longitudinal retrospective approach. This study is the first known attempt to determine the correlation between the TyG index and mortality in a rural Chinese population with MetS. However, the present study had some limitations. First, the participants were from one province in Northeast China, limiting the generalizability of the findings. Second, the TyG index is based on a single blood test, which could introduce potential bias. Third, even after controlling for potential confounding variables, residual confounding factors might have endured. Finally, the influence of prescribed medications on triglyceride and glucose levels could have affected the findings.

## Conclusion

The TyG index is a prominent risk predictor of all-cause mortality in participants with MetS in rural China. Our findings indicated that this simple and inexpensive index facilitates the early prediction of mortality in individuals with MetS, aiding village doctors in stratifying high-risk participants and implementing timely interventions.

## Data Availability

Data is available upon the reasonable request of the corresponding author.

## Abbreviations

BMI: body mass index
CV: cardiovascular
CVD: cardiovascular disease
FPG: fasting plasma glucose
HDL-C: high-density lipoprotein cholesterol
HIEG: hyperinsulinemic euglycemic
HOMA-IR: homeostatic model assessment for insulin resistance
IR: insulin resistance
LDL-C: low-density lipoprotein cholesterol
MetS: metabolic syndrome
TyG: triglyceride-glucose
